# Development, Optimization, and Evaluation of Nano Self-Emulsifying Drug Delivery System Formulation Platform for Oral Bioavailability Enhancement of Sulfasalazine and Disulfiram in Lung Cancer Chemoprevention

**DOI:** 10.1007/s11095-025-03964-7

**Published:** 2025-12-16

**Authors:** Preshita Desai, Katherine Bang, Mohammed Riaz Hasan Chowdhury, Zhijun Kevin Wang, Jeffrey Wang, Sunil Prabhu, Xueqing Liang, Fekadu Kassie

**Affiliations:** 1 Department of Biotechnology and Pharmaceutical Sciences, College of Pharmacy, Western University of Health Sciences, Pomona, CA 91766-1854, USA; 2 Department of Clinical Pharmacy Practice, School of Pharmacy & Pharmaceutical Sciences, University of California, Irvine, CA 92697, USA; 3 College of Veterinary Medicine, University of Minnesota, Saint Paul, MN 55108, USA; 4 Masonic Cancer Center, University of Minnesota, Minneapolis, MN 55455, USA

**Keywords:** bioavailability, disulfiram, lung cancer chemoprevention, nano-SEDDS, sulfasalazine

## Abstract

**Objective:**

Lung cancer chemoprevention modalities are gaining wide attention as it is the second most diagnosed cancer type and the leading cause of cancer-related deaths. Our previous studies reported unique lung cancer chemoprevention capability with a repurposed drug combination of sulfasalazine (SAS) and disulfiram (DSF). However, their efficacy is limited by poor bioavailability. To overcome this challenge, we developed bioenhanced oil-in-water (o/w) nano self-emulsifying drug delivery system (Nano-SEDDS) formulations of SAS and DSF.

**Methods:**

Unique isotropic Nano-SEDDS of SAS and DSF were developed and optimized using a single-step mix method followed by *in vitro* physicochemical characterization and stability studies. An *in vivo* pharmacokinetic and tissue-biodistribution study was undertaken to test the proposed hypothesis of bioavailability enhancement with Nano-SEDDS of SAS and DSF.

**Results:**

The optimal Nano-SEDDS formulation exhibited low nanodroplet sizes (< 200 nm), high drug content, and 4.5-fold (*p* < 0.01) and 3.75-fold (*p* < 0.01) enhancement in *in vitro* dissolution of SAS and DSF compared to the respective free drugs. The Nano-SEDDS formulations were also confirmed to be stable at room temperature in compliance with ICH guidelines. Further, SAS Nano-SEDDS showed a dose-dependent increment in oral bioavailability as shown by a significant 7.9-fold (*p* < 0.0001) enhancement in dose-normalized AUC at a dose of 10 mg/kg compared to free drug treatment at a control dose of 250 mg/kg.

**Conclusion:**

Overall, the studies corroborated the successful formulation of bioavailability-enhanced SAS and DSF Nano-SEDDS with future co-delivery applications for lung cancer prevention.

## Introduction

Lung cancer is the second most diagnosed cancer type and the leading cause of cancer-related mortality, with ~ 125,000 casualties in the USA (~ 20% cancer-related deaths) in 2025 [1]. These statistics are alarming despite tremendous efforts focused on advanced treatment modalities, lifestyle modifications, and more sensitive early diagnostic tools. This rationalizes the need for early intervention with effective and safe chemopreventive measures that can significantly mitigate lung cancer. Lifestyle changes like a balanced diet, smoking cessation, exercise etc. have been beneficial in cancer prevention, but more active and potent strategies like cancer chemoprevention are gaining wider attention in recent years. Specifically, cancer chemoprevention is defined as use of natural, synthetic, and biological drug/products or their combinations to block, delay, or reverse carcinogenesis [2, 3].

Earlier, we identified a repurposed drug combination of sulfasalazine (SAS) and disulfiram (DSF) to elicit unique lung cancer chemoprevention efficacy [4]. Both drugs are USFDA-approved with proven safety, wherein SAS (an anti-inflammatory medication) is clinically used in the treatment of rheumatoid arthritis, ulcerative colitis, etc., and DSF is used to overcome alcoholism problems.

We have reported that the combination of SAS-DSF can critically modulate oxidative stress-related pathways, inhibiting lung cancer and cancer stem cells (CSCs). Etiologically, tobacco smoking-induced genetic-epigenetic alterations are the key contributors, accounting for ~ 90% of lung cancer cases. Though tobacco smoke affects all types of cells in the lungs, its impact on stem cells is the most critical owing to its high mutation/alteration proclivity. Selective inhibition of these altered CSCs could be a groundbreaking strategy for chemoprevention of lung cancer [4]. Further, Specifically, our studies specifically demonstrated that DSF abrogated SAS-induced NRF2, xCT, and ALDH1A1 expression showing strong lung cancer chemoprevention potential [4].

Though the SAS-DSF combination has potent lung cancer chemoprevention efficacy with proven safety (USFDA-approved and being repurposed), its poor bioavailability limits the *in vivo* efficacy. [4-9]. SAS is a biopharmaceutical classification system (BCS) class IV drug, representing very poor solubility and permeability, leading to limited *in vivo* drug absorption and very poor oral bioavailability of 10–30% [4-6]. DSF belongs to BCS class II (very poor solubility but high permeability), indicating that DSF bioavailability directly depends on the drug solubility, as it is the only rate-limiting factor for *in vivo* drug absorption [7-9]. Hence, we aimed to develop bioavailability-enhanced SAS and DSF nanoformulations to ensure potent *in vivo* lung cancer prevention.

Specifically, a strategy for bioavailability enhancement of SAS needed both enhancement in drug solubility and permeation, while for DSF, enhancement in drug solubility was a primary requirement. Considering the ultimate objective of co-delivery of SAS and DSF formulations, it was rationalized to use the same nanoformulation platform to encapsulate both drugs enabling future mixing of drug formulations at a desired dose combination. In this context, as both SAS and DSF are lipophilic, the use of lipid-based nanocarriers was rationalized as they can better encapsulate small lipophilic drugs, rendering bioavailability enhancement. Further, the lipid nanocarriers encapsulate the drugs and allow enhanced permeation and absorption owing to their nano size. Literature and our prior experience with lipid nanocarriers have established enhanced bioavailability for a multitude of lipophilic drugs, including SAS and DSF [10-17].

**Fig. 1:**
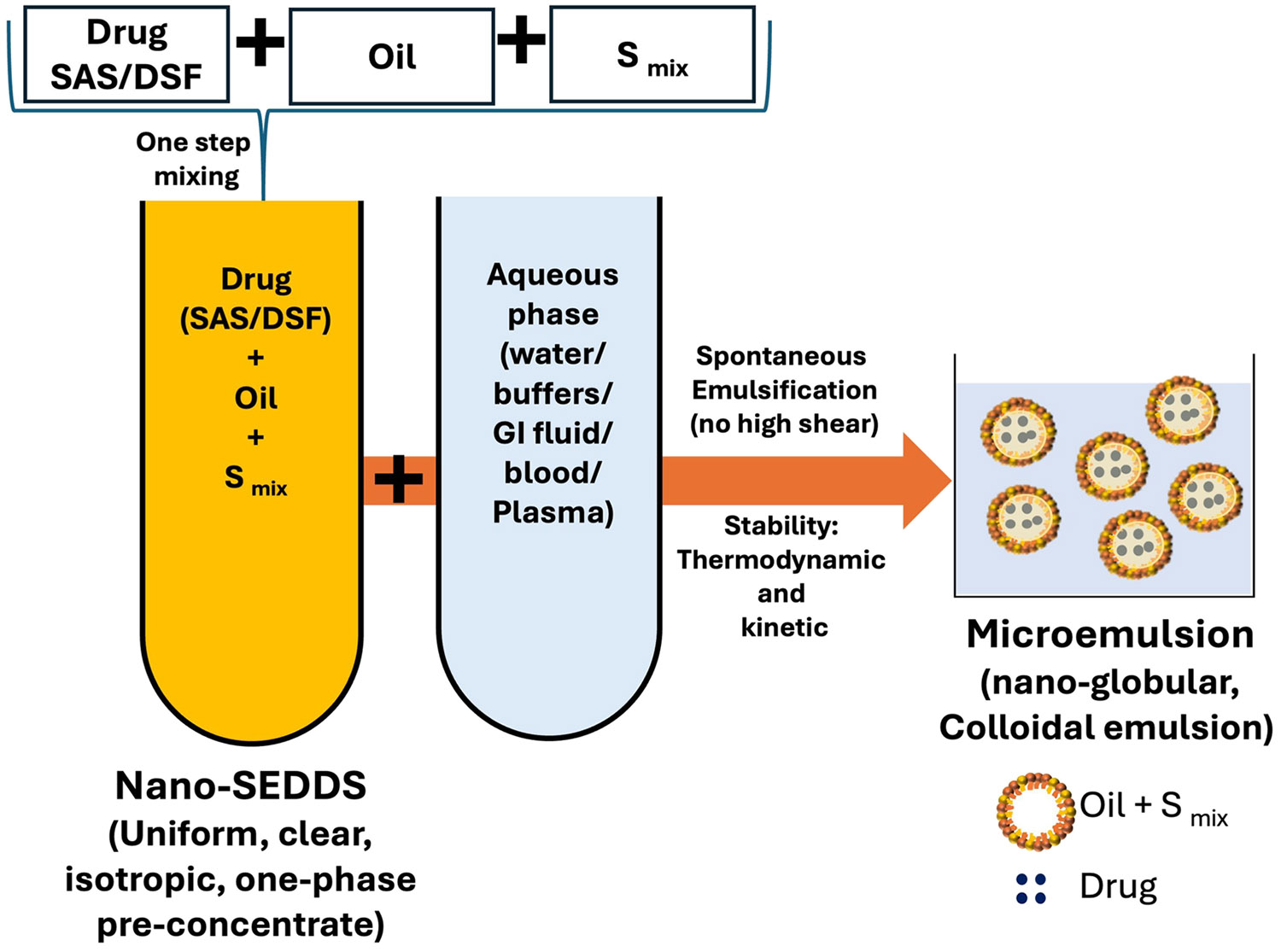
Schematic representation of SAS Nano-SEDDS and DSF Nano-SEDDS formulation development

Specifically, we rationalized the use of oil in water (o/w) nano self-emulsifying drug delivery system (Nano-SEDDS) as they are 1. unique, simple, and stable preconcentrates comprising isotropic mixture of oil/s with surfactant/s and/or stabilizer/s. They undergo spontaneous emulsification in the presence of an aqueous phase, forming a nano-colloidal emulsion (microemulsion) with superior kinetic and thermodynamic stability. Therefore, Nano-SEDDS formulations are preferred over the traditional biphasic emulsions (require multi-step formulation process and face stability challenges of biphasic systems) as they are formulated with a simple one-step mixing process to form a stable, monophasic isotropic pre-concentrate which, upon oral administration, can undergo spontaneous microemulsification with gastrointestinal (GI) fluid, forming a nano-globular emulsion; 2. capable of incorporating hydrophobic drugs forming supersaturated product; 3. capable of enhancing drug dissolution and passive targeted absorption due to nano-colloidal size, resulting in bioenhancement; 4. one-step simple mix method (Fig. 1) preparation allows easy scalability with stability demonstrating commercial translation viability [18, 19].

In this study, Nano-SEDDS formulations of SAS and DSF were developed and optimized, followed by detailed *in vitro* physicochemical characterization and stability studies. Further, an *in vivo* pharmacokinetic and tissue-biodistribution study was undertaken to test the proposed hypothesis of bioavailability enhancement with lipid Nano-SEDDS of SAS and DSF.

## Materials and Methods

### Materials

SAS (Millipore Sigma Inc., USA), DSF (Millipore Sigma Inc., USA), sulfapyridine (SFP, Millipore Sigma Inc., USA), methyl diethyldithiocarbamate (MDD, Millipore Sigma Inc., USA), diphenhydramine (DPH, Millipore Sigma Inc., USA), Tween 80 (T80, Sigma Chemicals Inc., USA), polyethylene glycol 400 (PEG 400, Alfa Aesar Inc., USA), propylene glycol (PG, Alfa Aesar Inc., USA), Spectra/Por^®^ Dialysis membrane-MWCO:50 kD (Spectrum, Labs Inc., USA), sodium taurocholate (Spectrum, Labs Inc., USA), lecithin (Spectrum, Labs Inc., USA), pepsin (Spectrum, Labs Inc., USA), maleic Acid (Spectrum, Labs Inc., USA), ammonium acetate (Spectrum, Labs Inc., USA), sodium chloride (Spectrum, Labs Inc., USA), hydrochloric acid (Spectrum, Labs Inc., USA), sodium hydroxide (Spectrum, Labs Inc., USA) were purchased. Transcutol HP (THP, Gattefosse Ltd., France) and Capmul MCM C8 (CC8, Abitec Corp., USA) were received as gift samples.

### Formulation Development and Optimization of SAS Nano-SEDDS and DSF Nano-SEDDS

The SAS and DSF Nano-SEDDS were developed individually to enhance bioavailability, but with an ultimate objective of co-delivery of SAS and DSF nanoformulations for lung cancer prevention. To enable future co-delivery, we carefully selected a unique excipient composition of Nano-SEDDS capable of encapsulating both drugs. This approach would allow their future mixing at various ratios to identify an optimal dose combination to effectively prevent lung tumorigenesis and cancer progression while retaining their nanoformulation physicochemical properties.

Nano-SEDDS comprises a unique isotropic mixture of oil/s with surfactant/s- stabilizer/s at a specific weight ratio. For this, preformulation solubility studies of SAS and DSF in various oils (data not shown) were conducted, and CC8 was selected as an optimum oily phase as it showed solubility for both drugs (SAS and DSF). Our previous studies have established the significant bioavailability enhancement with CC8-based Nano-SEDDS [11, 12]. As a first step, previously reported formulation of CC8 Nano-SEDDS [CC8 and surfactant mix (S_mix_) comprising T80 and THP] was used as a platform technology to incorporate SAS and DSF, forming SAS Nano-SEDDS and DSF Nano-SEDDS, respectively [11, 12]. The reformulated SAS Nano-SEDDS and DSF Nano-SEDDS were stable and formed colloidal nanosized emulsion upon mixing with water. However, the Nano-SEDDS represented a very low drug loading of 0.2% w/w and 0.5% w/w for SAS and DSF, respectively. To address this challenge, it was rationalized to further optimize the formulation to enhance drug loading.

For this, based on the high solubility of both DSF and SAS, PEG 400 and PG were shortlisted and explored as additional stabilizers/solubilizers. To identify the optimum composition of the Nano-SEDDS water titration method was used. Specifically, isotropic mixtures of oil (CC8) and S_mix_ (T80-THP-PEG 400 and T80-THP-PG) at 1:9 to 9:1 were prepared by vortexing. To this, water was added dropwise to identify visual turbidity point. If no turbidity was observed at 100X dilution, the Nano-SEDDS were considered to form a nano-colloidal microemulsion. The observed turbidity points were plotted using a pseudo-ternary phase diagram, and the microemulsion region was identified. The pseudo-ternary phase diagrams were compared to identify a composition resulting in a higher microemulsion area, and the optimized composition of Nano-SEDDS was selected. Further, drug loading studies were conducted, wherein the drug SAS (0.2–2%) and DSF (0.5 –2%) were loaded into blank Nano-SEDDS to form a clear, isotropic one-phase system. The saturation drug loading was identified as the highest concentration of SAS or DSF, which resulted in a clear-isotropic formulation devoid of any drug precipitation. All studies were performed in triplicate.

### Physicochemical Characterization of Optimized SAS Nano-SEDDS and DSF Nano-SEDDS

Unless otherwise mentioned, all characterization studies were conducted in triplicate (*n* = 3), and the data were reported as mean ± S.D. Where applicable, the quantification of SAS and DSF was conducted using an in-house developed and validated analytical HPLC method.

#### Appearance and Clarity

The SAS Nano-SEDDS and DSF Nano-SEDDS, and the microemulsion formed after 100X dilution with purified water, were observed for color, clarity, and precipitate formation. Further, the isotropic nature of the formulations was assessed by measuring the transmittance at 650 nm with a UV spectrophotometer (Shimadzu, USA).

#### Globule Size, Polydispersity Index, and Zeta Potential

The formulations were individually subjected to 100X dilution with purified water. The globule size, polydispersity index (PDI), and zeta potential were measured with Zetasizer Nanoseries Nano-ZS90 set at 4 mW standard laser at 633 nm with scattered light detection at 90° scattering angle.

#### Effect on pH and Dilution

The SAS Nano-SEDDS and DSF Nano-SEDDS were individually diluted (100X and 1000X) using purified water, pH 1.2 buffer, and pH 7.4 buffer to understand the combined effect on pH and dilution on the formulation. Each sample was subjected to globule size, PDI analysis (Sect. "Globule size, Polydispersity Index, and Zeta Potential"), and transmittance analysis (Sect. "Appearance and Clarity").

#### Drug Content Assay

SAS Nano-SEDDS and DSF Nano-SEDDS formulations equivalent to 10 mg of SAS and DSF were accurately weighed, followed by dissolution in 10 mL of methanol. The methanolic solutions were appropriately diluted and quantified for SAS and DSF using an in-house validated HPLC method.

#### *In Vitro* Drug Release Study

At an early stage of development, *in vitro* dissolution studies were performed using a discriminatory dissolution medium (Phase 1) as a fast and reliable model to assess the enhancement in dissolution profile of SAS Nano-SEDDS and DSF Nano-SEDDS compared to the free drug, respectively. Specifically, SAS (10 mg), SAS Nano-SEDDS (equivalent to 10 mg of SAS), DSF (10 mg), and DSF Nano-SEDDS (equivalent to 10 mg of DSF) were accurately placed in the dialysis bag and sealed with clips (donor compartment). The dialysis bags were individually housed in a dissolution apparatus (37 ± 0.5 °C and a stirring speed of 100 rpm) containing 900 mL of pH 7.4 phosphate buffer with 1% T80 (discriminatory dissolution medium). At each time point, samples (5 mL) were withdrawn from the dissolution apparatus, and the volume was replaced with a fresh dissolution medium. The samples were centrifuged (5000 rpm, 5 min) and the SAS and DSF concentration was quantified using an in-house developed HPLC method. Statistical analysis was performed using an unpaired t-test with significance at p-value ≤ 0.05 (GraphPad Prism software, USA).

Furthermore, to better predict the *in vivo* performance of the developed nanoformulations upon oral administration, it was also important to assess and compare the *in vitro* dissolution profile of the developed nanoformulations in a biorelevant dissolution medium for a longer duration that mimics the gastrointestinal physiological condition and formulation transit upon oral administration. Specifically, for this, the individual dialysis bags were prepared as the protocol described above and were individually housed in a dissolution apparatus (37 ± 0.5 °C and a stirring speed of 100 rpm) containing FaSSGF (fasted state simulated gastric fluid) for 1 h, followed by FaSSIF (fasted state simulated intestinal fluid) for an additional 4 h. FASSGF and FASSIF were prepared as per the composition described in the literature [20]. At each time point, samples (5 mL) were withdrawn from the dissolution apparatus, and the volume was replaced with a fresh dissolution medium. The samples were processed and analyzed using a similar protocol as above.

### SAS Nano-SEDDS and DSF Nano-SEDDS Stability Studies in Compliance with ICH Guidelines

The formulation stability study was executed per the recommendations of the International Council for Harmonization of Technical Requirements of Pharmaceuticals for Human Use (ICH) stability guidelines for storing the drug products at room temperature [21]. Specifically, the SAS Nano-SEDDS and DSF Nano-SEDDS contained in the amber glass bottles were subjected to storage at accelerated stability conditions (40 °C ± 2 °C/75% RH ± 5% RH) for 6 months; intermediate stability conditions (30 °C ± 2 °C/65% RH ± 5% RH) for 1 year and long-term stability conditions (25 °C ± 2 °C/60% RH ± 5% RH) for 1 year. At predetermined time intervals, samples were collected from storage and were subjected to various key physicochemical parameter analyses that included appearance (Sect. "Appearance and Clarity"), globule size, PDI, and zeta potential (Sect. "Globule size, Polydispersity Index, and Zeta Potential"), drug content assay (Sect. "Drug Content Assay"), and *in vitro* drug release (Sect. "In vitro Drug Release Study").

### *In Vivo* Studies

The *in vivo* pharmacokinetic and lung tissue-biodistribution study protocol was approved by the Institutional Animal Care and Use Committee of the University of Minnesota, USA (Protocol # 2206-40135A).

Female A/J mice were obtained from Jackson Lab, USA, and 3 mice/time point/dose group were randomly assigned to SAS and DSF groups. Before receiving oral treatment, mice were acclimatized to laboratory conditions (food and water *ad libitum)*. For SAS treatment, the animals received the free drug at 250 mg/kg (control dose) while the SAS Nano-SEDDS at 3 doses, viz., 10 mg/kg (low dose), 20 mg/kg (medium dose), and 30 mg/kg (high dose). For DSF treatment, the animals received the free drug at 100 mg/kg (control dose) while the DSF Nano-SEDDS at 3 doses, viz., 5 mg/kg (low dose), 10 mg/kg (medium dose), and 20 mg/kg (high dose). Blood and the lung tissue were harvested at the specified time points of 30 min, 1 h, 2 h, 4 h, 8 h, and 24 h post-administration. The blood samples were subjected to plasma separation by centrifugation at 2000 × *g* for 15 min at 4 °C. Both plasma and lung tissue samples were stored at −80 °C until analysis. An In-house developed LC–MS/MS method was employed to quantify the drugs and drug metabolites from the samples.

#### Development of LC–MS/MS Bioanalytical Method

The method was developed to quantify SAS and its metabolite SFP. DSF could not be quantified using the LC–MS/MS method as it is extremely unstable in plasma [9]. Therefore, only its metabolite MDD was quantified using LC–MS/MS analysis [22, 23]. DPH was used as an internal standard (IS). Specifically, the LC–MS/MS comprised API 3200 LC–MS/MS system and Shimadzu LC-20AD Prominence Liquid Chromatograph pumps. The chromatographic separation was performed using a Zorbax SB C18 column (150 × 2.1 mm, 5 μm) with an SB-C_18_ guard cartridge (12.5 × 2.1 mm). The isocratic elution was performed using a mobile phase of acetonitrile and 0.1% formic acid (80:20, v/v) at a flow rate of 0.3 mL/min. Typical mass spectrometric conditions were gas 1, nitrogen (40 psi); gas 2, nitrogen (40 psi); ion spray voltage, 5000 V; ion source temperature, 450 °C; curtain gas, nitrogen (25 psi). Multiple reaction monitoring (MRM) scanning in positive ionization mode was used to monitor the transitions of the analytes.

Methanolic stock solutions (1 mg/mL SAS and SFP; 10 mg/mL MDD) were serially diluted to get working solutions ranging from 20 to 5000 ng/mL for SAS and SFP, and 500 to 50,000 ng/mL for MDD. The internal standard (IS) was prepared by diluting the DPH stock solution to 1000 ng/mL concentration using 50% methanol. Calibration standard samples were prepared by spiking 10 μL of the standard working solutions into 100 μL of blank plasma to generate concentrations ranging from 2 to 500 ng/mL for SAS, SFP, and 50 to 5000 ng/mL for MDD.

The plasma samples were processed directly, while the tissue samples were homogenized thoroughly before extraction. Briefly, ~ 100 mg tissue samples were mixed with 3 × (by volume) cold saline, followed by homogenization at ~ 10,000 rpm for 30 s. The sample extraction protocol was developed after trying multiple methods in-house. Specifically, the plasma samples and lung tissue homogenate for SAS and SFP were extracted using the solid phase extraction method using the Oasis HLB cartridge. The cartridge was conditioned with 1 mL methanol and washed with 1 mL 0.05 M EDTA. To 100 μL of plasma sample, 10 μL of IS solution was added followed by mixing with 100 μL of 5% ammonia. The mixture was loaded onto the HLB cartridge and washed with 1 mL 1% acetonitrile. The samples were then eluted with 1 mL acetonitrile, and 10 μmL of the supernatant was injected for analysis. The plasma and lung tissue homogenate of MDD were extracted using a protein precipitation method. Briefly, 10 μL of IS working solution and 50% methanol were added to 100 μL of plasma sample. 300 μL of acetonitrile (1% acetic acid) was added to the mixture and vortexed for at least 1 min. The mixture was centrifuged at 10,000 rpm for 5 min, and 10 μL of the supernatant was injected for analysis. Based on quantification, pharmacokinetic and biodistribution profiles resulting from control drug treatment and nanoformulations were determined.

Non-compartmental analysis (NCA) was performed using PKanalix (Lixoft, France) to evaluate the pharmacokinetic parameters of SAS and SFP. Plasma concentration–time data were analyzed to calculate the area under the concentration–time curve from time zero to the last quantifiable concentration (AUC_0_–t), peak drug concentration (C_max_), and time to peak drug concentration (T_max_). The elimination rate constant (λz) was determined using log-linear regression, and T_1/2_ was calculated as ln [2] /λz. The below the lower limit of quantification (BLQ) samples at 24 h were treated as 1 ng/mL for regression (50% of BLQ). All analysis was conducted in triplicate, and the results were calculated as mean ± SD. The data were compared for statistical significance by the one-way analysis of variance (ANOVA) followed by post-hoc analysis (Bonferroni test) using Graph Pad InStat software (Graph Pad Software Inc., CA, USA).

## Results and Discussion

### Formulation Development and Optimization of SAS Nano-SEDDS and DSF Nano-SEDDS

**Fig. 2:**
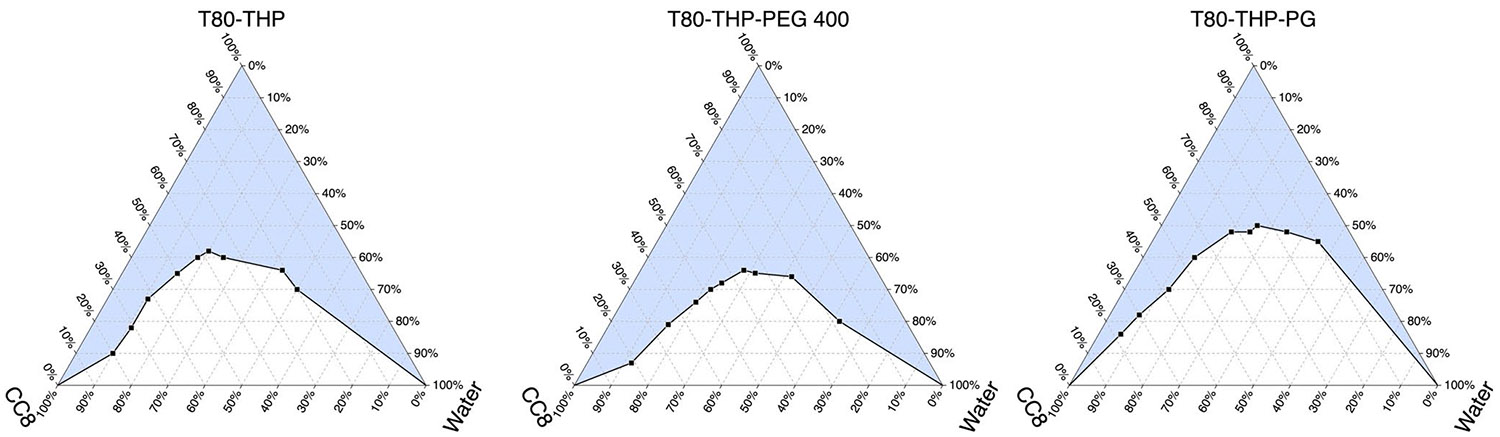
Pseudo-ternary phase diagram representing the microemulsion region for Nano-SEDDS prepared with different S_mix_. (CC8: Capmul MCM C8, T80: Tween 80, THP: Transcutol HP, PEG 400: polyethylene glycol 400, PG: propylene glycol)

The pseudo-ternary phase diagrams for various Nano-SEDDS prepared with CC8 as a constant oily phase while altering the S_mix_ composition are represented in Fig. 2. All pseudo-ternary phase diagrams confirmed the formation of thermodynamically and kinetically stable microemulsions without high shear but represented variation microemulsion regions. Specifically, the pseudo-ternary phase diagram with S_mix_ containing T80-THP was used as a reference because, during preliminary studies, SAS and DSF both were successfully incorporated into this Nano-SEDDS system, and the objective of this optimization study was to enhance drug loading with additional solvent/stabilizer, viz. PEG 400 or PG. Upon comparison, it was observed that the pseudo-ternary phase diagram with S_mix_ containing T80-THP-PEG 400 showed almost similar microemulsion area to the S_mix_ containing T80-THP. The pseudo-ternary phase diagram with S_mix_ containing T80-THP-PG showed a smaller microemulsion area than the S_mix_ containing T80-THP. PEG 400 is a hydrophilic polyethylene glycol polymer with a molecular weight of ~ 400 Da. It is reported to interfere with the strong hydrogen bond in the aqueous phase thereby enhancing the dissolution of hydrophobic drugs (BCS class II and IV) and has superior co-surfactant-like properties [24-26]. In our opinion, the superior co-surfactant nature of PEG 400 worked in favor of Nano-SEDDS stabilization upon emulsification. PG is a strong solvent for hydrophobic drugs, but due to its smaller size, it has limited co-surfactant-like properties because though it can enhance the interfacial fluidity, it tends to reduce the polarity of water [27]. The addition of PG to the nano-SEEDS compromised the stabilization of Nano-SEDDS marginally resulting in a smaller microemulsion region in the phase diagrams compared to reference.

Therefore, Nano-SEDDS comprising CC8 and S_mix_ containing T80-THP-PEG 400 were considered optimum and were tested for drug-loading studies. For SAS and DSF, saturation drug loading up to 0.9% w/w and 1.4% w/w was achievable forming stable, isotropic Nano-SEDDS devoid of any drug precipitation respectively. Therefore, with the use of PEG 400 in the Nano-SEDDS, SAS and DSF saturation drug loading could be enhanced by 4.5-fold and threefold respectively.

This enhancement in drug loading confirmed the successful development and optimization of one Nano-SEDDS platform for delivery of both SAS and DSF. Though it was possible to use drug-saturated SAS and DSF nano-SEEDS, considering the intended low dose of both drugs for lung cancer management coupled with bioavailability-enhanced nanoformulations, it was rationalized to use the sub-saturated SAS and DSF drug-loaded Nano-SEDDS. The use of drug sub-saturated Nano-SEDDS has the advantages of long-term stability without drug precipitation due to lower than saturation loading along with better drug encapsulation and absorption. With this in consideration, the final optimized formulation was prepared with 0.6% w/w drug loading for SAS Nano-SEDDS and 0.8% w/w drug loading for DSF Nano-SEDDS. The optimized formulations were subjected to detailed physicochemical characterization.

### Physicochemical Characterization of Optimized SAS Nano-SEDDS and DSF Nano-SEDDS

The quantification of SAS and DSF was conducted using an in-house developed and validated analytical HPLC method using an Agilent 1260 Infinity II system. Chromatographic separation of SAS quantification was achieved using XTerra RP18 column (150 X 4.6 mm, 5 μm) using a mobile phase comprising methanol: ammonium acetate buffer (80%: 20%; pH 7.0) at a flow rate of 1.2 mL/min with an injection volume of 20 μL and a detection wavelength of 360 nm. The method was observed to be linear over a concentration range of 2–50 μg/mL (r^2^ = 0.9999) with adequate sensitivity, with a limit of detection (LOD) and a limit of quantification (LOQ) of 0.15 μg/mL and 0.5 μg/mL, respectively. The intraday accuracy and precision (% recovery: 98–101, % RSD: < 1) and interday accuracy and precision (% recovery: 96–101, % RSD: < 1.2) indicated excellent recovery and reliability. Chromatographic separation of DSF was achieved using Phenomenex Luna C18 column (150 X 4.6 mm, 5 μm) using a mobile phase comprising methanol:water (80%: 20%) at a flow rate of 1 mL/min with an injection volume of 20 μL and a detection wavelength of 275 nm. The method was observed to be linear over a concentration range of 5–50 μg/mL (r^2^ = 0.9999) with adequate sensitivity, with a LOD and LOQ of 0.3 μg/mL and 1 μg/mL, respectively. The intraday accuracy and precision (% recovery: 96–102, % RSD: < 1.4) and interday accuracy and precision (% recovery: 98–102, % RSD: < 1.1) indicated excellent recovery and reliability.

**Fig. 3:**
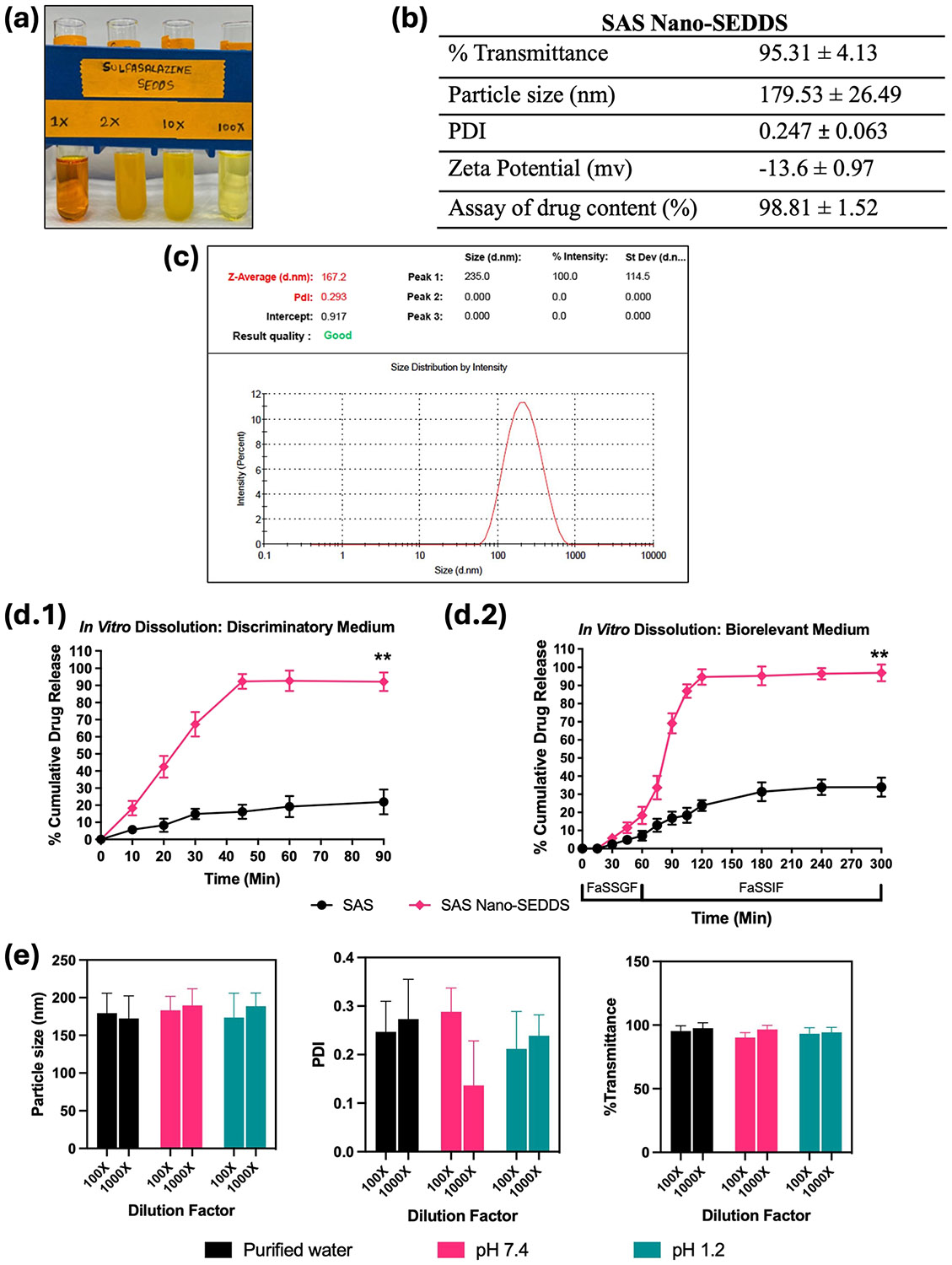
Physicochemical characterization of SAS Nano-SEDDS. Where applicable, data are presented as mean ± SD (*n* = 3). (**a)** Image of SAS Nano-SEDDS formulation preconcentrate and upon dilution with water. (**b)** Various physicochemical parameters of SAS Nano-SEDDS (**c**) Representative particle size distribution plot for SAS Nano-SEDDS obtained using Zetasizer Nanoseries Nano-ZS90. (**d)** *In vitro* drug dissolution profile of SAS as free drug and SAS Nano-SEDDS using dialysis bag method in (d.1) discriminatory dissolution medium and (d.2) biorelevant dissolution medium. Statistical significance was analyzed by unpaired t-test (statistical significance represented as * *p* < 0.05, ** *p* < 0.01; *** *p* < 0.001; **** *p* < 0.0001). (**e**) Effect of pH and dilution on SAS Nano-SEDDS

The SAS Nano-SEDDS preconcentrate was observed to be a dark yellow translucent isotropic solution [Fig. 3(a)] which underwent spontaneous emulsification upon dilution with purified water forming a stable light yellow microemulsion. Drug SAS is yellow which resulted in the yellow color to the final product. The results of various *in vitro* physicochemical analyses of SAS Nano-SEDDS are summarized in Fig. 3. The SAS Nano-SEDDS exhibited a very high transmittance of 95.31 ± 4.13%. Such high transmittance is possible only when the light passes through the formulation without scattering, which in turn confirms the product clarity and isotropicity. Further, the SAS Nano-SEDDS size was observed to be 179.53 ± 26.49 nm (PDI: 0.247 ± 0.063). Such low nano-globule size is desired not only for the formation of microemulsion resulting in enhanced rate and extent of solubilization but will also enhance the permeation and in turn the bioavailability. The low PDI value represents a uniform globule size with a narrow size distribution confirming the formation of stable and uniform product [refer to Fig. 3(c) for a representative size distribution plot]. Together, the low globule size, spontaneous emulsification, clear, isotropic, and stable nature of SAS Nano-SEDDS confirmed the formation of the microemulsion. The zeta potential of SAS Nano-SEDDS was calculated to be −13.6 ± 0.97 mV. The excipients used in Nano-SEDDS formulation are non-ionic and the observed mild negative zeta potential can be explained by the ionization of SAS which is a weak acid with a pKa of 2.65 [28]. Based on the SAS Nano-SEDDS formulation development study (Sect. "Formulation Development and Optimization of SAS Nano-SEDDS and DSF Nano-SEDDS"), the SAS drug loading in the nanoformulation was optimized at a sub-saturated concentration of 0.6% w/w. To ensure complete incorporation, stability, and uniform distribution of SAS, an assay of SAS drug content in the nanoformulation was conducted. The assay results showed a SAS drug content of 98.81 ± 1.52%, confirming successful drug loading and uniform distribution. SAS belongs to BCS class IV with hallmarks of low solubility and low permeability. SAS-Nano-SEDDS formulation was strategized because of its unique properties to form spontaneous drug-dissolved microemulsion which can achieve enhanced drug dissolution. *In vitro* drug release studies (Phase 1) was performed using a discriminatory dissolution medium for quick and reliable assessment of the dissolution profile of SAS Nano-SEDDS compared to free drug and the results are depicted in Fig. 3(d.1). SAS Nano-SEDDS exhibited over 90% drug release within 45 min followed by a plateau up to 90 min which was ~ 4.5 times higher than free drug SAS (p Value < 0.01) over the same study period. Additionally, the rate of dissolution of SAS Nano-SEDDS was significantly higher compared to the free drug SAS. In Phase 2, longer and detailed *in vitro* drug release studies were performed using a biorelevant dissolution medium mimicking the gastrointestinal physiological condition (using FaSSGF, followed by FaSSIF) as a better predictor of *in vivo* performance of nanoformulation upon oral delivery. The results are depicted in Fig. 3(d.2). The dissolution studies in simulated gastric fluid (FaSSGF) for SAS Nano-SEDDS showed limited dissolution of SAS (18.34 ± 4.73%), but it was ~ 2 times higher compared to free SAS at the end of 1 h. This initial limited dissolution of free SAS can be explained as SAS is a weak acid with very low solubility in acidic pH as observed in the case of simulated gastric fluid (FaSSGF). Further, SAS Nano-SEDDS potentially enhanced the SAS drug dissolution in FaSSGF by spontaneous emulsification and solubilization of the drug to some extent. Continued dissolution in simulated intestinal fluid (FaSSIF) with higher pH (pH 6.5) showed a steep increase in the rate and extent of SAS dissolution, with SAS nano-SEDDS reaching over 95% in the next 120 min, followed by a plateau, which was ~ 3 times higher than free drug SAS (p-value < 0.01). Further, in addition to increased pH high concentration of sodium taurocholate, lecithin (surfactant/stabilization effect) in SaSSIF compared to SaSSGF should also have contributed to the higher dissolution [20]. Overall, the enhanced rate and extent of dissolution with SAS Nano-SEDDS can be explained by the formation of isotropic microemulsion that incorporated the BCS class IV drug SAS into emulsified nano globules (179.53 ± 26.49 nm) rendering fast and enhanced solubilization. Our earlier studies with Nano-SEDDS and other literature evidence corroborate the finding and unique capacity of Nano-SEDDS to enhance drug dissolution [10, 11, 17, 26, 27].

As discussed earlier, upon dilution with an aqueous phase, Nano-SEDDS undergo spontaneous emulsification, to form nano-globules. Under multiple *in vitro* and *in vivo* conditions, variations in pH and/or dilution can critically influence the spontaneous emulsification process, thereby impacting the size, stability, and other physicochemical properties of Nano-SEDDS. Hence, it was imperative to investigate and understand the impact of these parameters on Nano-SEDDS to optimize formulation performance and confirm consistent nano-drug delivery outcomes. Specifically, particle size, PDI, and % transmittance calculated after diluting the SAS Nano-SEDDS with purified water, buffer pH 7.4, and buffer pH 1.2 at varied ratios are depicted in Fig. 3 (e). Overall, with variation in pH or dilution of aqueous phase, the SAS Nano-SEDDS did not show any precipitation or significant change in the assessed parameters indicating the stability and robustness of the Nano-SEDDS formulation platform. This ensured the formation of uniform nano-globules under various *in vitro* and/or *in vivo* conditions.

**Fig. 4:**
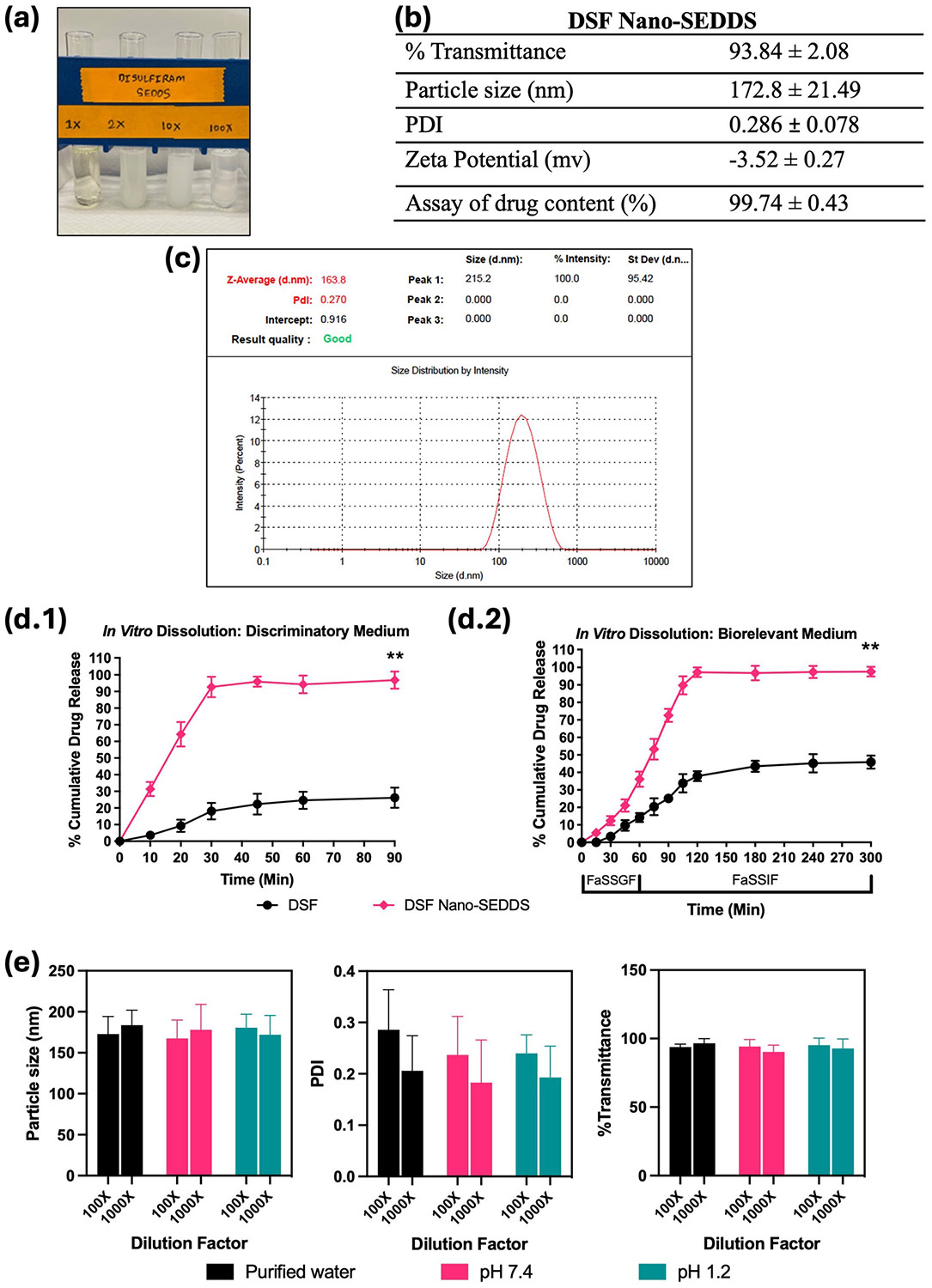
Physicochemical characterization of DSF Nano-SEDDS. Where applicable, data is presented as mean ± SD (*n* = 3). (**a**) Image of DSF Nano-SEDDS formulation preconcentrate and upon dilution with water. (**b**) Various physicochemical parameters of DSF Nano-SEDDS. (**c**) Representative particle size distribution plot for DSF Nano-SEDDS obtained using Zetasizer Nanoseries Nano-ZS90. (**d**) *In vitro* drug dissolution profile of DSF as free drug and DSF Nano-SEDDS using dialysis bag method in (d.1) discriminatory dissolution medium and (d.2) biorelevant dissolution medium. Statistical significance was analyzed by unpaired t-test (statistical significance represented as * *p* < 0.05, ** *p* < 0.01; *** *p* < 0.001; **** *p* < 0.0001). (**e**) Effect of pH and dilution on SAS Nano-SEDDS

The DSF Nano-SEDDS preconcentrate was observed to be a clear-buff colored translucent isotropic solution (Fig. 4) which underwent spontaneous emulsification upon dilution with purified water forming a stable microemulsion. The results of various *in vitro* physicochemical analyses of SAS Nano-SEDDS are summarized in Fig. 4. Drug DSF is white while all excipients are clear to light buff liquids which resulted in the color of the final product. The DSF Nano-SEDDS showed a very high transmittance of 93.84 ± 2.08% confirming product clarity and isotropicity. The particle size of DSF Nano-SEDDS was 172.8 ± 21.49 nm with a PDI of 0.286 ± 0.078 [refer to Fig. 4 (c) for representative size distribution plot]. The observed nano-globule size with a narrow size distribution, product clarity, and isotropicity confirmed the formation of uniform and stable microemulsion. Further, the optimized excipient matrix used to formulate both SAS and DSF was the same and the studies confirmed that both drugs could be successfully incorporated into the said Nano-SEDDS resulting in uniform low nano-globular size allowing future mixing of both Nano-SEDDS for codelivery of SAS and DSF (data not shown and SAS-DSF mixed Nano-SEDDS for co-delivery were used for *in vivo* efficacy studies which is not within the scope of this paper). The zeta potential of DSF Nano-SEDDS was calculated to be −3.52 ± 0.27 mV. The excipients used in the Nano-SEDDS formulation are non-ionic which explains the very low zeta potential. Such low zeta potential may reflect a possible instability due to enhanced coagulation; therefore, steric stabilizers like T80 and PEG 400 were selected to ensure formulation stability [11, 24, 29, 30]during formulation development. Based on the DSF Nano-SEDDS formulation development study (Sect. "Formulation Development and Optimization of SAS Nano-SEDDS and DSF Nano-SEDDS"), 0.8% w/w DSF drug loading was optimized. To ensure complete incorporation, stability, and uniform distribution of DSF, an assay of DSF drug content in the nanoformulation was conducted. The assay results showed a DSF drug content of 99.74 ± 0.43, confirming successful drug loading and uniform distribution. DSF belongs to BCS class II with a hallmark of low solubility but good permeability. Hence, the formulation objective was to enhance the apparent solubility of DSF with DSF Nano-SEDDS which combined with already reported good permeability would enhance the absorption and bioavailability of DSF *in vivo*.

*In vitro* drug release studies (Phase 1) was performed using a discriminatory dissolution medium and the results are depicted in Fig. 4(d.1). DSF Nano-SEDDS exhibited over 90% drug release within 30 min reaching over 95% within 45 min followed by a plateau up to 90 min which was ~ 3.75 times higher than the free drug DSF (p Value < 0.01) over the same study period. Additionally, DSF Nano-SEDDS showed over tenfold faster drug dissolution within the first 10 min, confirming the faster rate of drug dissolution with DSF Nano-SEDDS compared to free drug DSF. The results of Phase 2 *in vitro* drug release studies (longer and detailed study using a biorelevant dissolution medium) are depicted in Fig. 4(d.2). The dissolution studies in simulated gastric fluid (FaSSGF) for DSF Nano-SEDDS showed 36.16 ± 4.38% at the end of 1 h reaching over 95% in the next 60 min (total 2 h), which was ~ 2 times higher than free drug DSF (p-value < 0.01). Overall, the Nano-SEDDS triggered spontaneous emulsification of DSF dissolved nano-globules that resolved the critical challenge of DSF’s low solubility, confirming the hypothesis of enhanced drug solubilization with the Nano-SEDDS platform. Further, high concentration of sodium taurocholate, lecithin (surfactant/stabilization effect) in SaSSIF compared to SaSSGF should also have contributed to the higher rate of dissolution in the later phase [20].

Further, the effect of variation in pH and dilution on DSF Nano-SEDDS was assessed by calculating particle size, PDI, and % transmittance after diluting the formulation with purified water, buffer pH 7.4, and buffer pH 1.2 at varied ratios is depicted in Fig. 4(e). At all dilutions, no precipitate was observed as confirmed with visual observation, and % transmittance data and formulation showed a non-significant change in particle size and PDI with variation confirming the stability of DSF Nano-SEDDS towards various aqueous media and confirming the strong emulsification potential of the meticulously selected excipient composition.

#### SAS Nano-SEDDS and DSF Nano-SEDDS Stability Studies in Compliance with ICH Guidelines

**Fig. 5:**
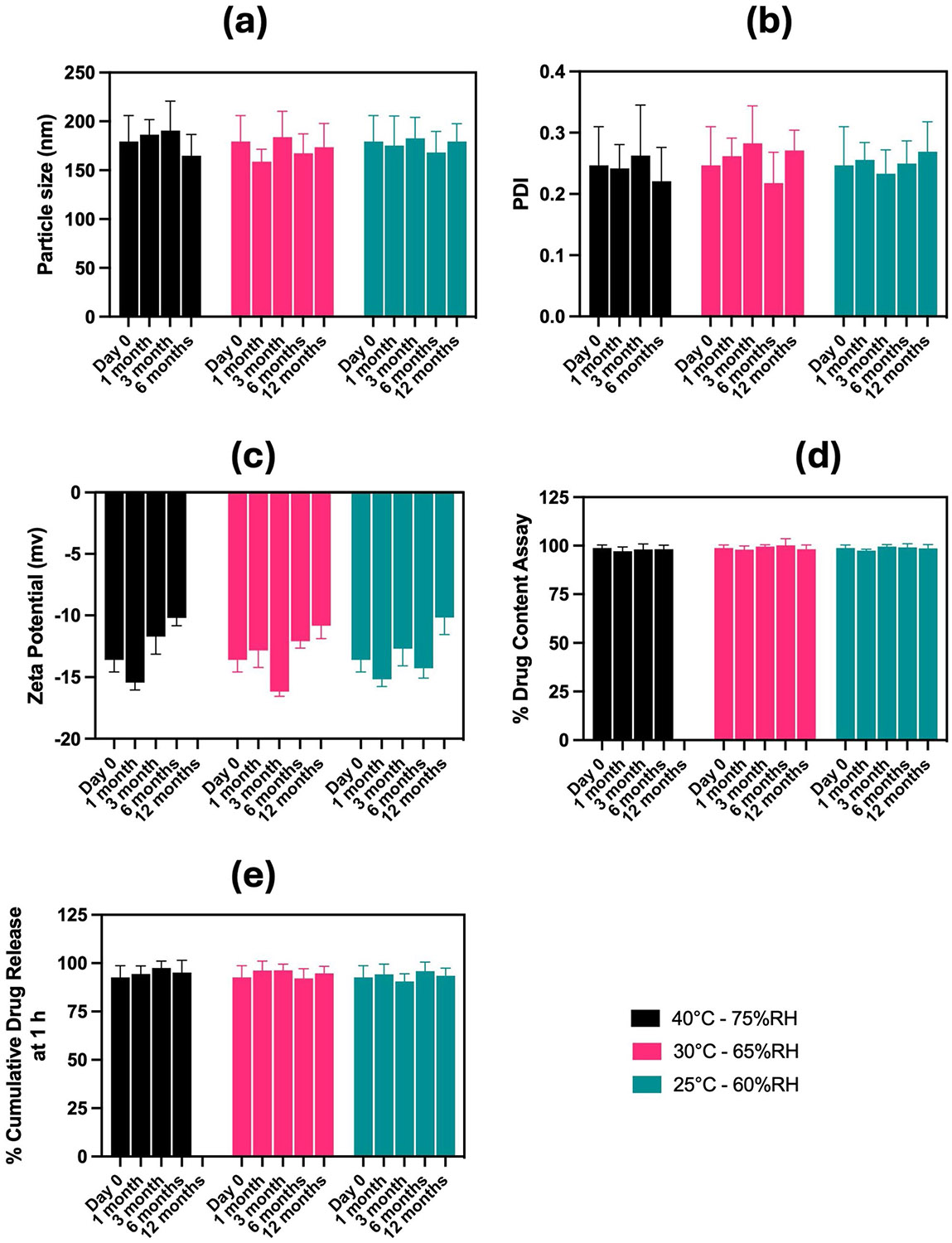
Results of stability study testing of SAS Nano-SEDDS per ICH guidelines. Data presented as mean ± SD, n = 3. Analysis of (**a**) Particle size, (**b**) Polydispersity index (PDI), (**c**) Zeta potential, (**d**) % drug content assay, and (**e**) % cumulative drug release at 1 h

**Fig. 6:**
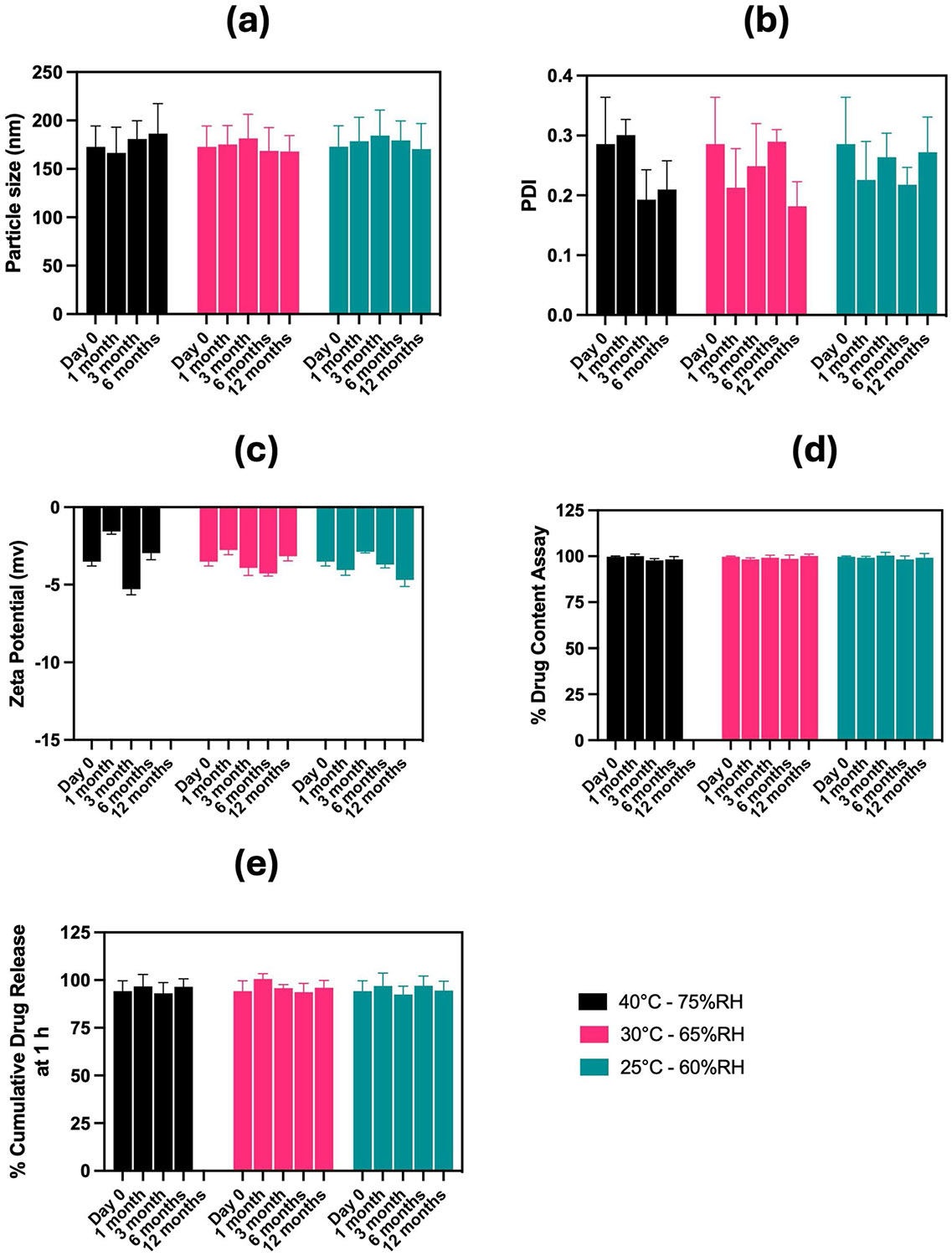
Results of stability study testing of DSF Nano-SEDDS per ICH guidelines. Data presented as mean ± SD, n = 3. Analysis of (**a**) Particle size, (**b**) Polydispersity index (PDI), (**c**) Zeta potential, (**d**) % drug content assay, and (**e**) % cumulative drug release at 1 h

The stability studies were executed in agreement with the ICH stability guidelines for drug products intended for storage at room temperature. Further, the baseline physicochemical characterization of both SAS Nano-SEDDS and DSF Nano-SEDDS, as discussed in Section "Physicochemical characterization of Optimized SAS Nano-SEDDS and DSF Nano-SEDDS", was used to establish the initial (Day 0) formulation parameters. These served as a reference for assessing the stability of the drug and formulation throughout the stability study. The formulation characterization results for the stability samples of SAS Nano-SEDDS and DSF Nano-SEDDS are presented in Figs. 5 and 6 respectively.

Throughout the stability testing period, the SAS Nano-SEDDS retained its yellow translucent appearance, with no significant changes in particle size, PDI, zeta potential, or drug content assay. All parameters were reported to be well within the confidence interval of the in-house specified acceptable limits relative to the Day 0 reference value (Fig. 5). This confirmed the stability of both drug SAS and the Nano-SEDDS formulation, ensuring the retention of spontaneous emulsification, which led to the formation of drug-dissolved nano-droplets with uniform size and narrow size distribution.

The % cumulative drug release at 60 min was considered a reference point, as initial *in vitro* drug release studies conducted during optimized formulation characterization [Sect. "Formulation Development and Optimization of SAS Nano-SEDDS and DSF Nano-SEDDS", Fig. 3(d)] indicated that maximum drug release was achieved, with a plateau observed at this time point. At predetermined stability testing time points, the % cumulative drug release at 60 min was calculated and compared against the Day 0 reference value of 92.7 ± 6% SAS cumulative release. The results indicated that storage samples did not deviate significantly (remaining within a 10% variation) from the reference value for % cumulative drug release at 60 min, confirming that the formulation retained its dissolution profile, thus confirming product stability.

Similarly, DSF Nano-SEDDS retained its clear-buff translucent appearance and exhibited no significant variation in particle size, PDI, zeta potential, drug content, or % cumulative drug release at 60 min. All observations remained well within the confidence interval of the in-house specified acceptable limits relative to the Day 0 reference value (Fig. 6). These results confirmed the stability of DSF Nano-SEDDS over the stability testing period.

To summarize, based on the stability testing data, both SAS Nano-SEDDS and DSF Nano-SEDDS can be successfully stored at room temperature without any indication of formulation performance failure. In our opinion, the Nano-SEDDS platform plays a vital role in ensuring product stability during storage because it comprises a unique combination of oils, surfactants, and/or stabilizers specific to the given drug, which are uniformly mixed to form an isotropic oily pre-concentrate (devoid of an aqueous phase). As a result, during storage, Nano-SEDDS can be visualized as a simple oily solution, and as a known fact, solutions inherently have superior thermodynamic and kinetic stability [11]. From a formulation performance standpoint, it is possible to prepare and store Nano-SEDDS products as a monophasic oily preconcentrate because of their unique feature to spontaneously emulsify (without any high shear or rigorous mixing) upon exposure to an aqueous phase (either upon consumption or dilution), forming a drug-dissolved nano-droplet emulsion. This emulsification process enhances drug saturation solubility and dissolution, while the smaller droplet size facilitates improved permeation. Further, the use of sub-saturation concentration for SAS and DSF for drug loading also played a role in product stability. As the drug loading was less than the saturation solubility of Nano-SEDDS, (Refer to Sect. "Formulation Development and Optimization of SAS Nano-SEDDS and DSF Nano-SEDDS"), it significantly reduced the chances of drug precipitation and enhanced the robustness of the product to withstand unfavorable temperature and humidity conditions witnessed under accelerated and long-term stability environment.

Overall, the Nano-SEDDS product, prepared and stored as an oily pre-concentrate, not only ensures product storage stability (mitigating the commonly reported instability issues of otherwise prepared and stored emulsions) but also guarantees *in-situ* spontaneous emulsification for better product performance. Overall, we believe that this formulation strategy is uniquely suited to ensure both product stability and performance.

#### *In Vivo* Studies

A robust and reproducible LC–MS/MS bioanalytical method was developed for the quantification of SAS, its metabolite SFP, and DSF and its metabolite MDD. MRM scanning in positive ionization mode was used to monitor the transitions of the analytes. The specific mass spectrometric conditions were identified which are shown in Table 1. The developed bioanalytical method was robust with good linearity and sensitivity. DSF could not be quantified using the LC–MS/MS method as it is extremely unstable in plasma, and hence, only its metabolite MDD was quantified. The standard curve demonstrated good linearity for concentrations ranging from 2 to 500 ng/mL for SAS and SFP and from 50 to 5000 ng/mL for MDD. The accuracy of the method ranged between 85 and 115%. The lower limit of quantification (LLOQ) for plasma samples was determined as 2 ng/mL for SAS and SFP and 50 ng/mL for MDD.

The treatment was satisfactorily tolerated by all the animals in all treatment groups at various doses as confirmed by healthy behavior with no adverse reaction. Specifically for this, mice were observed, between treatment and sacrifice, for changes in appearance and behavior (physical state, activity, gait, grooming etc.), autonomic signs (change in the size of the pupil, nasal and ocular discharge, piloerection and salivation), gastrointestinal symptoms (food and water consumption, nature of feces, urination etc.), neurological signs (sensory changes, seizure etc.), and respiratory and cardiovascular system alterations (breathing, changes in the color of mucous membranes). Monitoring for these symptoms did not reveal any treatment-related alterations.

**Fig. 7:**
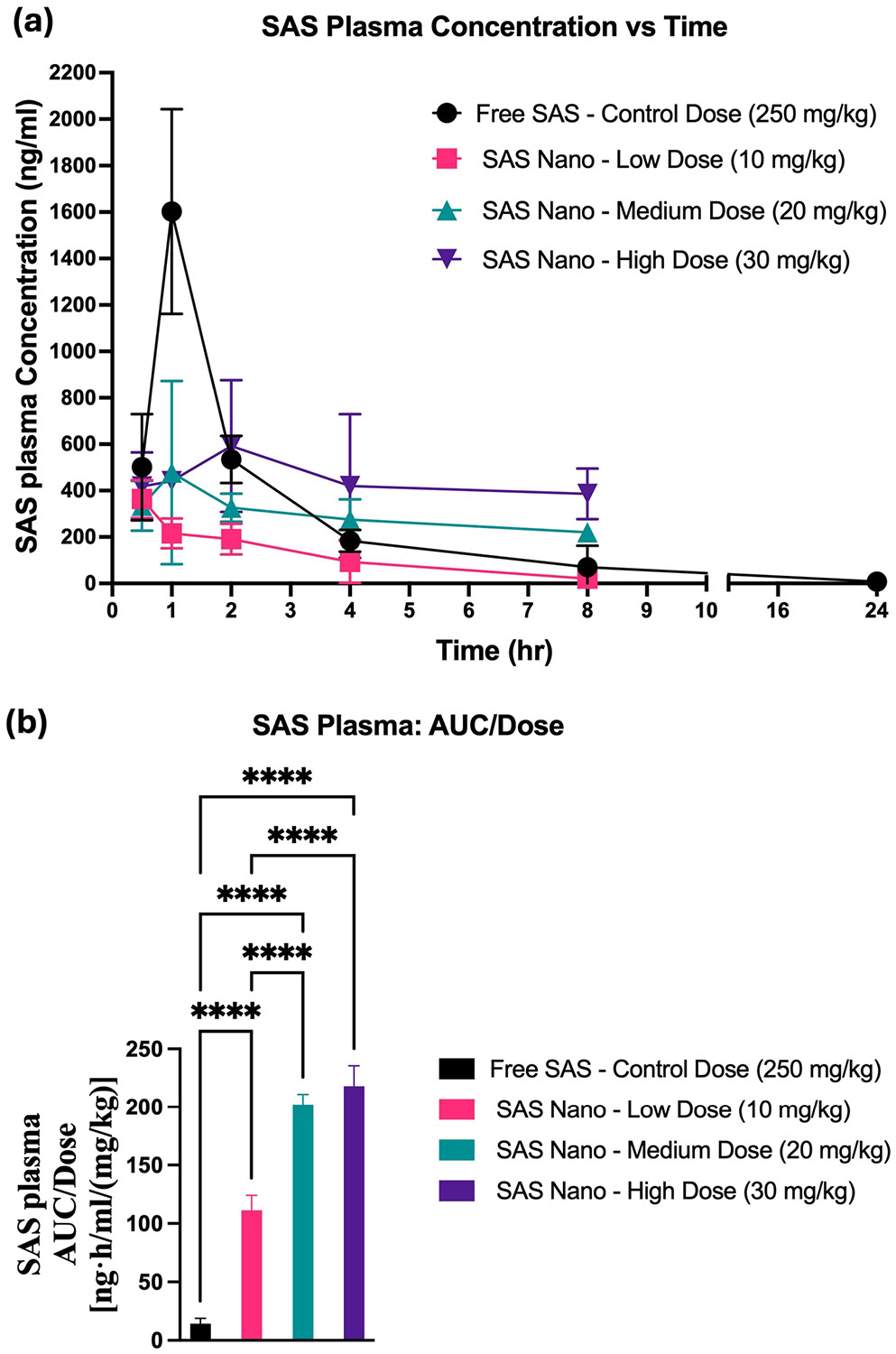
*In vivo* pharmacokinetic performance of SAS upon oral treatment with free drug SAS and SAS Nano-SEDDS at various doses in female A/J mice. (**a**) Plasma concentration vs time profile of SAS. (**b**) Dose normalized AUC represented as AUC/dose. Data presented as mean ± SD, n = 3. Statistical significance was analyzed by one-way ANOVA followed by *post-hoc* analysis using Bonferroni’s test (statistical significance represented as * *p* < 0.05, ** *p* < 0.01; *** *p* < 0.001; **** *p* < 0.0001)

**Fig. 8:**
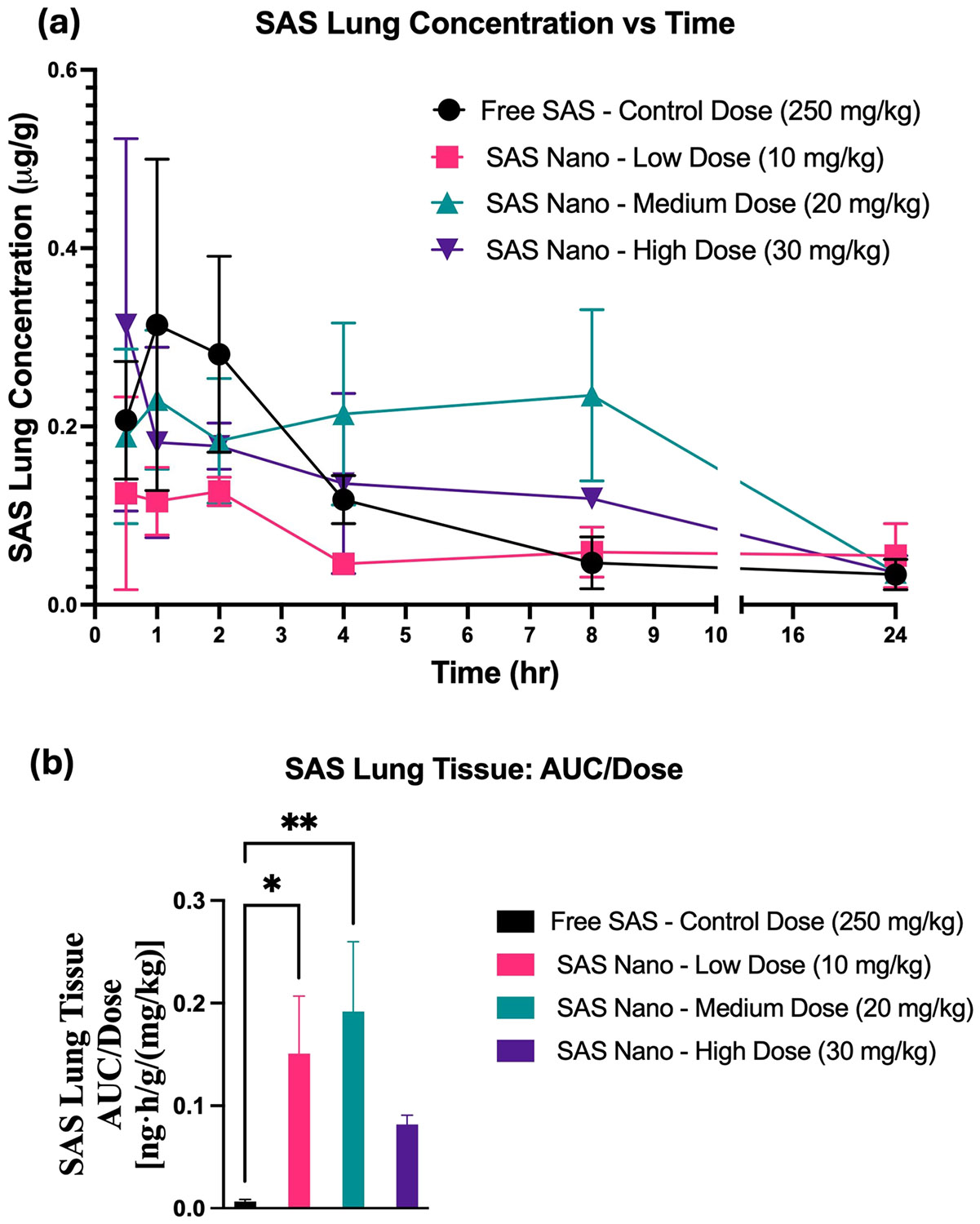
*In vivo* lung tissue-biodistribution performance of SAS upon oral treatment with free drug SAS and SAS Nano-SEDDS at various doses in female A/J mice. (**a**) Tissue concentration vs time profile of SAS. (**b**) Dose normalized tissue AUC represented as AUC/dose. Data presented as mean ± SD, *n* = 3. Statistical significance was analyzed by one-way ANOVA followed by post-hoc analysis using Bonferroni’s test (statistical significance represented as * *p* < 0.05, ** *p* < 0.01; *** *p* < 0.001; **** *p* < 0.0001)

For SAS, the plasma concentrations *versus* time pharmacokinetic profile and lung tissue-biodistribution profile following standard free drug treatment and SAS Nano-SEDDS formulation treatment at 3 doses are shown in Figs. 7 and 8, respectively. Various pharmacokinetic and biodistribution parameters were calculated, and the results were dose-normalized considering the variation in the dose of each treatment. The Pharmacokinetic and biodistribution parameter comparison of SAS is presented in Tables 2 and 3, respectively.

The plasma concentration vs time pharmacokinetic profile indicated significant enhancement in the bioavailability of SAS when administered as SAS Nano-SEDDS formulation [(Fig. 7(a)]. Only SAS elimination T_1/2_ could be calculated accurately due to the limited samples points. Although the SAS t_1/2_ Nano-SEDDS groups appeared to be shorter than control (*P* < 0.05, 1-way ANOVA), the results were not consistent among different dose levels. One potential source of error could be the use of 50% BLQ for BLQ samples at 24 h. Therefore, the results of t_1/2_ should be interpreted with caution and need to be further verified. The SAS Nano-SEDDS formulation at doses of 10 mg/kg (low dose), 20 mg/kg (medium dose), and 30 mg/kg (high) showed significant enhancement in dose-normalized relative AUC of 111.4 ± 12.6 ng.h/mL/(mg/kg) (7.9- fold; *p* < 0.0001), 202.1 ± 8.8 ng·h/mL/(mg/kg) (14.2-fold; *p* < 0.0001), and 218.1 ± 77.5 ng.h/mL/(mg/kg) (15.4-fold; *p* < 0.0001) compared to dose normalized relative AUC of 14.2 ± 4.7 ng·h/mL/(mg/kg) with free SAS treatment at very high control dose of 250 mg/kg [Table 2 and Fig. 7 (b)]. Further, the SAS Nano-SEDDS showed a dose-dependent increment in bioavailability up to the medium dose (20 mg/kg), followed by a non-significant increase in the dose-normalized AUC between the 20 mg/kg and 30 mg/kg doses (Table 2). This nonlinear dose-AUC/Dose relationship could result from SAS non-linear behavior at higher doses due to saturation of biological processes involved in absorption, distribution, metabolism, and excretion, and/or indirect metabolism by gut microbes, which are also reported in literature as the underlined reasons for SAS unpredictable bioavailability [31, 32]. Further, in the current proof-of-principle study, there exists a significant time gap between 8 and 24 h sample points. From a future study plan perspective, incorporation of additional time points between 8 and 24 h would allow for multiple point analysis and more detailed and accurate AUC calculation.

As the formulation is proposed for lung cancer prevention, the lung biodistribution analysis is a must. The lung tissue-biodistribution vs time profile indicated significant enhancement in the bioavailability of SAS when administered as the SAS Nano-SEDDS formulation compared to free SAS [(Fig. 8(a)]. The SAS Nano-SEDDS formulation at a dose of 10 mg/kg (low dose), 20 mg/kg (medium dose), and 30 mg/kg (high) showed significant enhancement in dose-normalized relative AUC of 0.151 ± 0.056 ng.h/g/(mg/kg) (21.6-fold; *p* < 0.05), 0.192 ± 0.068 ng.h/g/(mg/kg) (28.9-fold; *p* < 0.01), 0.082 ± 0.009 ng·h/g/(mg/kg) (11.7-fold) compared to dose normalized relative AUC of 0.007 ± 0.002 ng·h/g/(mg/kg) with free SAS treatment at very high control dose of 250 mg/kg [Table 3 and Fig. 8 (b)]. Further, the SAS Nano-SEDDS formulation significantly increased the SAS bioavailability as indicated by the dose normalized C_max_ and AUC, especially at higher doses up to 20 mg/kg [Table 3]. Surprisingly, at the highest dose of SAS Nano-SEDDS (30 mg/kg) indicated non-lienear tissue distribution profile with lower dose normalized relative AUC compared to medium dose (20 mg/kg). The exact reason for this observation is unknown but can be attributed to SAS nonlinear behavior at higher doses due to saturation of biological processes involved in absorption, distribution, metabolism, and excretion, and/or indirect metabolism by gut microbes, which are also reported in literature as the underlying reasons for SAS unpredictable bioavailability [31, 32]. As described earlier, we intend to repeat this investigation with multiple time points (especially between 8–24 h) window to have a clearer understanding and better comprehension of the data. Overall, this proof-of-principal investigation results confirm that the SAS Nano-SEDDS formulations significantly enhanced the rate and extent of absorption in blood and lung tissue uptake in a dose-dependent manner up to a SAS Nano-SEDDS dose of 20 mg/kg, enabling the treatment dose reduction by at least 25-fold.

This significant enhancement in bio-performance can be explained as follows. SAS belongs to BCS class IV representing very poor solubility and permeability leading to poor oral bioavailability. The meticulous use of the Nano-SEDDS formulation platform addresses both these challenges. As described earlier, Nano-SEDDS formulation forms colloidal nanodroplets in the GI track wherein the drug is solubilized using a unique combination of lipid-surfactant matrix hence addressing the challenge of low drug solubility [10, 11, 17]. Further, the nano droplet size of the Nano-SEDDS formulation allows better interaction and passive uptake resulting in enhanced permeability. Further, SAS is a substrate to efflux ATP-binding cassette (ABC) transporter, breast cancer resistance protein (BCRP)/ABCG2 expressed in multiple tissues including GI lining that are reported to be the rate-limiting step in SAS absorption. Tween 80 and PEG 400 excipients in SAS Nano-SEDDS formulation are known inhibitors of this efflux transporter. In our understanding, the SEDDS nanoformulation can further potentiate the bio-performance of SAS by inhibiting the drug efflux resulting in supplementary enhancement in drug absorption [25, 30, 33].

**Fig. 9:**
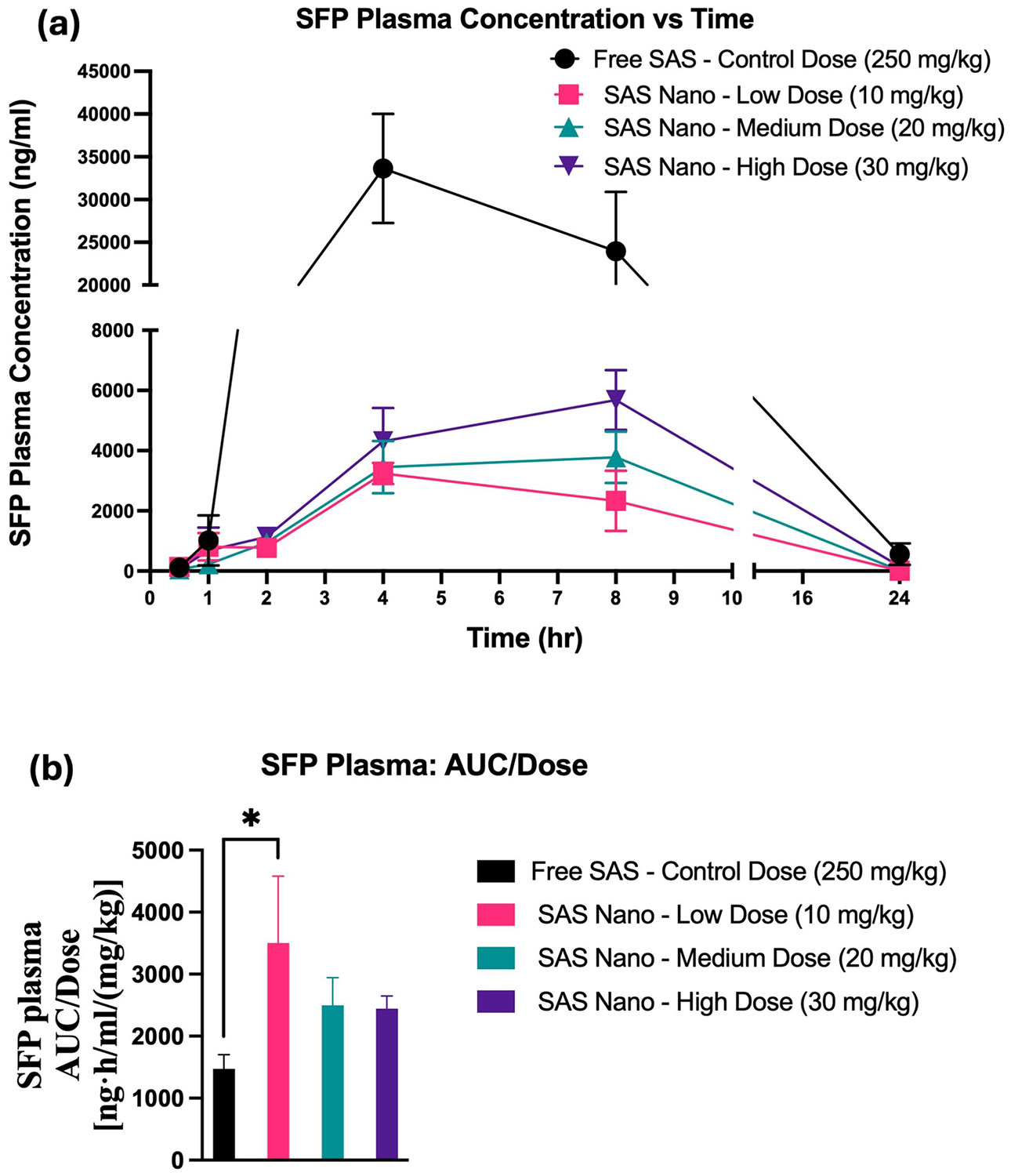
*In vivo* pharmacokinetic performance of SFP upon oral treatment with free drug SAS and SAS Nano-SEDDS at various doses in female A/J mice. (**a**) Plasma concentration vs time profile of SAS. (**b**) Dose normalized AUC represented as AUC/dose Data presented as mean ± SD, n = 3. Statistical significance was analyzed by one-way ANOVA followed by post-hoc analysis using Bonferroni’s test (statistical significance represented as * *p* < 0.05, ** *p* < 0.01; *** *p* < 0.001; **** *p* < 0.0001)

**Fig. 10:**
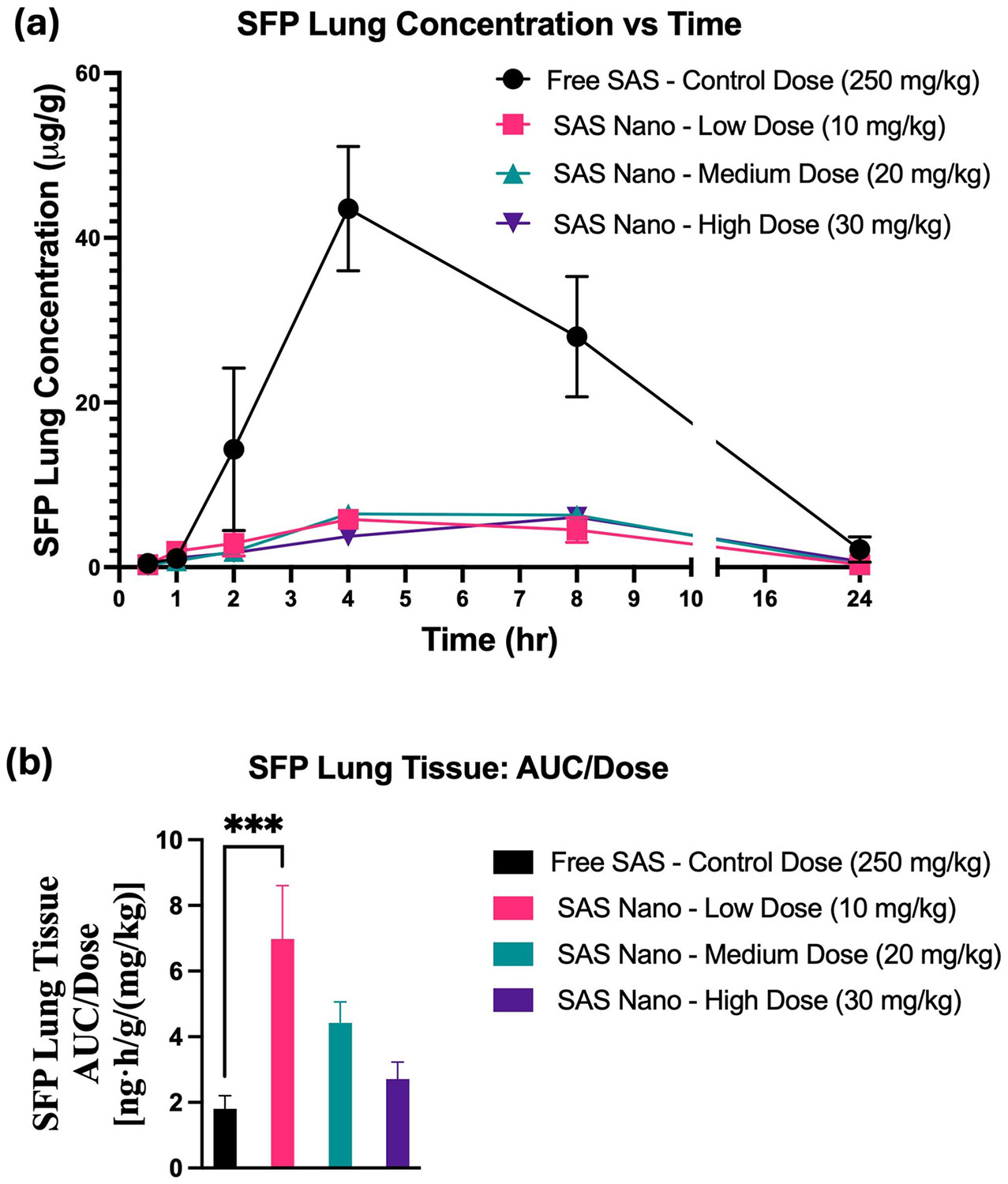
*In vivo* lung tissue-biodistribution performance of SFP upon oral treatment with free drug SAS and SAS Nano-SEDDS at various doses in female A/J mice. (**a**) Tissue concentration vs time profile of SAS. (**b**) Dose normalized tissue AUC represented as AUC/dose. Data presented as mean ± SD, *n* = 3. Statistical significance was analyzed by one-way ANOVA followed by post-hoc analysis using Bonferroni’s test (statistical significance represented as * *p* < 0.05, ** *p* < 0.01; *** *p* < 0.001; **** *p* < 0.0001)

SAS is fast metabolized to SFP (an anti-inflammatory agent) and hence the SFP pharmacokinetic and lung biodistribution profile was also analyzed to understand the SAS pharmacokinetics, metabolism, and retention. For SFP, the plasma concentrations *versus* time pharmacokinetic profile and lung tissue-biodistribution profile following standard free drug treatment and SAS Nano-SEDDS treatment at 3 doses are shown in Figs. 9 and 10 respectively. Various pharmacokinetic and biodistribution parameters were calculated, and the results were dose-normalized considering the variation in the dose of each treatment. The Pharmacokinetic and biodistribution parameter comparison of SFP is presented in Tables 4 and 5 respectively.

The plasma concentration vs time pharmacokinetic profile indicated slow metabolism of SAS with various treatment groups resulting in a gradual increase in SFP concentration with T_max_ achieved between 4–7 h (SAS pharmacokinetics achieved T_max_ 0.5–2 h; refer to Tables 2 and 4). Further, significant enhancement in the drug exposure of SFP when administered as SAS Nano-SEDDS formulation was reported [(Fig. 9(a)] with dose-dependent increment in AUC and C_max_ for SFP. [Table 4]. Further, even at 1/25th of the dose, the 10 mg/kg (low dose) SAS Nano-SEDDS formulation showed significant enhancement in SFP dose normalized AUC of 3500.6 ± 1082.5 ng.h/mL/(mg/kg) (2.37- fold; *p* < 0.05), compared to SFP dose normalized AUC of 1472 ± 2291 ng·h/mL/(mg/kg) with free SAS treatment at very high control dose of 250 mg/kg [Table 4 and Fig. 9 (b)]. The lung tissue-biodistribution vs time profile for SFP followed a similar course indicating significant high retention of SFP upon SAS Nano-SEDDS treatment at various doses compared to free drug SAS treatment. Interestingly, even at 1/25th of dose, the 10 mg/kg (low dose) SAS Nano-SEDDS formulation showed significant enhancement in SFP dose normalized AUC of 6.981 ± 1.62 ng·h/g/(mg/kg) (3.88-fold; *p* < 0.001), compared to dose normalized relative AUC of 1.8 ± 0.4 ng·h/g/(mg/kg) with free SAS treatment at very high control dose of 250 mg/kg [Table 5 and Fig. 10 (b)]. Overall, the results confirmed that the SAS Nano-SEDDS formulation significantly enhances the rate and extent of absorption in blood and lung tissue uptake in a dose-dependent manner, enabling the treatment dose reduction of at least 25-fold. Importantly, SAS Nano-SEDDS could increase the bioavailability of both SAS and SFP, but upon comparison of the AUC/D ratio of SAS and SFP, the ratio AUC/D of SAS is much higher (around tenfold) than the AUC/D ratio of SFP (around twofold). Hence, with SAS Nano-SEDDS, although the exposure of both SAS and SFP was increased, the increase in SAS bioavailability appeared to be more Significant.

As discussed earlier, DSF could not be quantified using the LC–MS/MS method as it is extremely unstable in plasma [9]. Therefore, only its metabolite MDD was quantified using LC–MS/MS analysis [22, 23]. Following a single administration of free drug DSF at 100 mg/kg (control dose) and the DSF Nano-SEDDS formulation at three doses, viz. 5 mg/kg (low dose), 10 mg/kg (medium dose), and 20 mg/kg (high dose), no detectable MDD concentration was observed. This could be attributed to the fast metabolism of DSF to MDD and unstable MDD in plasma and tissue samples [22]. We anticipate that a more sensitive quantitative method is required to detect MDD in plasma and tissue samples, which is the limitation of the current investigation. Although it was not possible to compare the pharmacokinetics and lung tissue-biodistribution of DSF nano SEDDS *vs.* free drug DSF, the significant enhancement in rate and extent of dissolution of DSF with nano-SEDDS (Refer to Section "Physicochemical characterization of Optimized SAS Nano-SEDDS and DSF Nano-SEDDS") is a promising proof-of-concept for overcoming the drug solubility challenge of DSF that directly impacts its bioavailability.

## Conclusion

Bioavailability-enhanced nanoformulations of repurposed drugs present a safe yet very effective modality for lung cancer prevention. The developed Nano-SEDDS platform showed superior formulation properties and stability. *In vivo* pharmacokinetic and tissue-biodistribution study confirmed the proposed hypothesis of bioavailability enhancement SAS lipid Nano-SEDDS. A more sensitive method for the analysis of DSF formulations will be the future scope of the investigation. As the next step, the SAS and DSF Nano-SEDDS were co-formulated and assessed for *in vivo* lung cancer chemoprevention efficacy, the results of which will be reported separately.

## Figures and Tables

**Table I:** Optimized Tandem Mass Conditions for LC–MS/MS Analysis of Analytes

Compounds	m/z transition	DP (V)	EP (V)	CEP (V)	CE (V)	CXP (V)
Sulfasalazine (SAS)	399–381	61.0	9.5	14.0	25.0	4.0
Sulfapyridine (SFP)	250–156	41.0	8.0	12.0	22.0	4.0
Methyl diethyldithiocarbamate (MDD)	164–116	26.0	6.0	14.0	15.0	4.0
Diphenhydramine (DPH, internal standard, IS)	256–167	22.0	5.0	15.0	22.0	4.0

DP: Declustering Potential; EP: Entrance Potential; CEP: Collision Entrance Potential; CE: Collision Energy; CXP: Cell Exit Potential

**Table 2:** Comparison of *in vivo* pharmacokinetic parameters of SAS upon oral treatment with free drug SAS and SAS Nano-SEDDS at various doses in female A/J mice (*n* = 3)

Pharmacokinetic Parameter	SAS Free drug	SAS Nano-SEDDS		
	Control 250 mg/kg	Low Dose 10 mg/kg	Medium Dose 20 mg/kg	High Dose 30 mg/kg
AUC (ng·h/mL)	3568.8 ± 1192.4	1114.8 ± 126.1	4043.4 ± 177	6545.1 ± 2326.2
C_max_ (ng/mL)	1602.1 ± 441.1	364.4 ± 81.9	545.9 ± 336.5	635.5 ± 241.8
T_max_ (h)	1 ± 0	0.5 ± 0	1.6 ± 0.5	1.1 ± 0.7
T_1/2_ (h)	3.61 ± 0.63	2.28 ± 0.33	3.27 ± 0.50	2.37 ± 0.13
AUC/Dose (ng·h/mL/(mg/kg))	14.2 ± 4.7	111.4 ± 12.6	202.1 ± 8.8	218.1 ± 77.5
C_max_/Dose (ng/mL/(mg/kg))	6.4 ± 1.7	36.4 ± 8.1	27.2 ± 16.8	21.1 ± 8

**Table 3:** Comparison of *in vivo* lung tissue-biodistribution parameters of SAS upon oral treatment with free drug SAS and SAS Nano-SEDDS at various doses in female A/J mice (*n* = 3)

Lung Biodistribution Parameter	SAS Free drug	SAS Nano-SEDDS		
	Control 250 mg/kg	Low Dose 10 mg/kg	Medium Dose 20 mg/kg	High Dose 30 mg/kg
AUC (ng·h/g)	1.86 ± 0.716	1.517 ± 0.568	3.84 ± 1.374	2.46 ± 0.281
C_max_ (ng/g)	0.369 ± 0.109	0.161 ± 0.075	0.264 ± 0.061	0.366 ± 0.132
T_max_ (h)	0.833 ± 0.288	1.166 ± 0.763	4.666 ± 3.055	1.666 ± 2.02
AUC/Dose (ng·h/g/(mg/kg))	0.007 ± 0.002	0.151 ± 0.056	0.192 ± 0.068	0.082 ± 0.009
C_max_/Dose (ng/g/(mg/kg))	0.001 ± 0	0.016 ± 0.007	0.013 ± 0.003	0.012 ± 0.004

**Table 4:** Comparison of *in vivo* pharmacokinetic parameters of SFP upon oral treatment with free drug SAS and SAS Nano-SEDDS at various doses in female A/J mice (*n* = 3)

Pharmacokinetic Parameter	SAS Free drug	SAS Nano-SEDDS		
	Control 250 mg/kg	Low Dose 10 mg/kg	Medium Dose 20 mg/kg	High Dose 30 mg/kg
AUC (ng·h/mL)	368020.7 ± 57261.1	35006.7 ± 10825.6	49976.3 ± 8925.3	73328.3 ± 6098.4
C_max_ (ng/mL)	34651.5 ± 4644.1	3243.1 ± 350.4	3990.8 ± 943.7	5831.2 ± 864.4
T_max_ (h)	5.3 ± 2.3	4 ± 0	6.6 ± 2.3	6.6 ± 2.3
AUC/Dose (ng·h/mL/(mg/kg))	1472 ± 229	3500.6 ± 1082.5	2498.8 ± 446.2	2444.2 ± 203.2
C_max_/Dose (ng/mL/(mg/kg))	138.6 ± 18.5	324.3 ± 35	199.5 ± 47.1	194.3 ± 28.8

**Table 5:** Comparison of *in vivo* lung tissue-biodistribution parameters of SFP upon oral treatment with free drug SAS and SAS Nano-SEDDS at various doses in female A/J mice (*n* = 3)

Lung Biodistribution Parameter	SAS Free drug	SAS Nano-SEDDS		
	Control 250 mg/kg	Low Dose 10 mg/kg	Medium Dose 20 mg/kg	High Dose 30 mg/kg
AUC (ng·h/g)	450.4 ± 121.7	69.818 ± 16.205	88.436 ± 12.834	81.354 ± 15.549
C_max_ (ng/g)	43.5 ± 7.5	5.403 ± 0.842	6.908 ± 0.585	6.08 ± 0.894
T_max_ (h)	4 ± 0	4.666 ± 3.055	5.333 ± 2.309	8 ± 0
AUC/Dose (ng·h/g/(mg/kg))	1.8 ± 0.4	6.981 ± 1.62	4.421 ± 0.641	2.711 ± 0.518
C_max_/Dose (ng/g/(mg/kg))	0.1 ± 0	0.54 ± 0.084	0.345 ± 0.029	0.202 ± 0.029

## Data Availability

The datasets generated during and/or analyzed during the current study are available from the corresponding author on reasonable request.
